# Effect of Thiouronium-Based Ionic Liquids on the Formation and Growth of CO_2_ (sI) and THF (sII) Hydrates

**DOI:** 10.3390/ijms23063292

**Published:** 2022-03-18

**Authors:** Mário R. C. Soromenho, Anastasiia Keba, José M. S. S. Esperança, Mohammad Tariq

**Affiliations:** Laboratório Associado para a Química Verde, REQUIMTE, Departamento de Química, Faculdade de Ciências e Tecnologia, Universidade NOVA de Lisboa, 2829-516 Caparica, Portugal; m.soromenho@fct.unl.pt (M.R.C.S.); a.keba@campus.fct.unl.pt (A.K.)

**Keywords:** CO_2_ hydrates, THF hydrates, ionic liquids, phase equilibria, nucleation onset temperature, hydrate growth

## Abstract

In this manuscript, two thiouronium-based ionic liquids (ILs), namely 2-ethylthiouronium bromide [C_2_th][Br] and 2-(hydroxyethyl)thiouronium bromide [C_2_OHth][Br], were tested at different concentrations (1 and 10 wt%) for their ability to affect CO_2_ (sI) and tetrahydrofuran (THF) (sII) hydrate formation and growth. Two different methods were selected to perform a thermodynamic and kinetic screening of the CO_2_ hydrates using a rocking cell apparatus: (i) an isochoric pressure search method to map the hydrate phase behavior and (ii) a constant ramping method to obtain the hydrate formation and dissociation onset temperatures. A THF hydrate crystal growth method was also used to determine the effectiveness of the ILs in altering the growth of type sII hydrates at atmospheric pressure. Hydrate–liquid–vapor equilibrium measurements revealed that both ILs act as thermodynamic inhibitors at 10 wt% and suppress the CO_2_ hydrate equilibria ~1.2 °C. The constant ramping methodology provides interesting results and reveals that [C_2_OHth][Br] suppresses the nucleation onset temperature and delays the decomposition onset temperatures of CO_2_ hydrates at 1 wt%, whereas suppression by [C_2_th][Br] was not statistically significant. Normalized pressure plots indicate that the presence of the ILs slowed down the growth as well as the decomposition rates of CO_2_ hydrates due to the lower quantity of hydrate formed in the presence of 1 wt% ILs. The ILs were also found to be effective in inhibiting the growth of type sII THF hydrates without affecting their morphology. Therefore, the studied thiouronium ILs can be used as potential dual-function hydrate inhibitors. This work also emphasizes the importance of the methods and conditions used to screen an additive for altering hydrate formation and growth.

## 1. Introduction

Gas hydrates are non-stoichiometric ice-like crystalline solids made up of gas molecules encapsulated within the polyhedral cavities formed by the hydrogen-bonded network of water [1]. The most common hydrate-forming molecules are gases—methane (CH_4_), propane (C_3_H_8_), butane (C_4_H_10_), carbon dioxide (CO_2_), nitrogen (N_2_), and hydrogen (H_2_)—of appropriate size to fit and stabilize the cages through van der Waals forces under conditions of low temperature and high pressures [2]. There are three main structures of hydrates depending on the size of the guest molecule, namely (i) cubic structure I (sI) formed by CH_4_, C_2_H_6_, and CO_2_; (ii) cubic structure II (sII) formed by C_3_H_8_ and iC_4_H_10_; (iii) hexagonal structure H (sH) generally formed by a binary mixture such as CH_4_+cyclopentane and CH_4_+neohexene [3].

Since there are no chemical bonds between the host and guest molecules involved in the formation of hydrates, the rates of formation and dissociation are relatively quick [4], which makes hydrate technology attractive for several applications such as gas capture and storage [5], transportation [6], gas separation [7], cold flow technology [8] or water desalination [9]. However, the limited solubility of some gases in water, the stochastic nature of the nucleation phenomena and the required conditions for hydrate formation are some of the challenges to overcome before taking any technology development to large-scale application [10].

The exploration of oil and gas is moving towards the deep sea, where conditions are more favorable for hydrates to form within the pipelines and flow assurance is a big concern for the oil and gas industries [11]. The hydrates formed not only hamper production by blocking the pipeline but the pressure build up due to a hydrate plug can be fatal [12]. A huge amount of money is generally spent to mitigate and manage the issue during production [13]. The scenario becomes challenging in the case of reservoirs, where the CO_2_ content is higher as it forms relatively stable hydrates in milder conditions compared to CH_4_. Moreover, CO_2_ is often injected in the wells for enhanced oil recovery, and therefore it is generally present there [14]. Some of the known natural gas wells where a high content of CO_2_ is present are located in the Central European Pannonian Basin, Sicily, North Sea South Viking Garben, South China Sea, Gulf of Thailand, Sarawak-Malaysia, Australian Cooper-Eromanga Basin and New Zealand [15].

Several methods exist in industry to address the hydrate formation issue during production, namely gas dehydration, pipeline insulation, depressurization and addition of chemical additives known as inhibitors, which is generally considered the most viable option [16]. Normally, methanol and ethylene glycol are used as thermodynamic hydrate inhibitors (THIs) because they shift the conditions of hydrate formation towards much lower temperature and high pressure. However, the amount required to shift the equilibrium could be as large as 25 wt%, which can affect the aquatic environment [17]. Therefore, in the last two decades, the development of new low-dosage hydrate inhibitors (LDHIs) that can be used in low quantities (<2 wt%) is on the rise [18]. LDHIs consist of two categories of inhibitors known as kinetic inhibitor (KHIs), which do not shift the conditions of hydrate formation but slow down the process, and anti-agglomerates (AAs), which allow hydrates to form as fine particles that remain dispersed in the liquid phase [19].

Ionic liquids (ILs), which are known for their unusual behavior, are low-melting salts consisting solely of cations and anions that can be tuned to obtain compounds with the desired set of properties and functionalities viz. physico-chemical properties, hydrophobicity, surface activity, biocompatibility, toxicity, and solubility [20,21,22,23]. ILs are used in numerous applications such as protein stabilization, amyloid inhibition, enhanced oil recovery, corrosion inhibition and desulfurization of fuels [24,25,26,27,28]. Pure ILs and, sometimes, blended versions with materials such as MOFs are used for CO_2_ capture and energy storage applications [29]. Recently, the potential of common and commercially available ILs to act as dual-function hydrate inhibitors and promoters was revealed [30].

The search for greener and low-dosage hydrate inhibitors is on the rise [18]. ILs, due to their designer nature, have the ability to fill this gap. The effect of ILs on CO_2_ hydrate formation and growth is relatively less explored compared to CH_4_ hydrates [31,32,33]. For instance, Makino et al. studied the effect of imidazolium-based ILs on CO_2_ hydrate phase behavior [34]. Cha et al. tested the effectiveness of morpholinium- and piperidinium-based ILs in inhibiting the CO_2_ hydrates [35]. Reports on the effect of ammonium-based ILs on the phase behavior of CO_2_ and mixed CO_2_ + CH_4_ hydrates are also available [36,37,38]. Apart from this, the effect of imidazolium- and ammonium-based ILs on the kinetics of CO_2_ and CO_2_ + CH_4_ mixed hydrate formation has also been reported [39,40,41]. Since only a handful of IL families are tested for their effectiveness on CO_2_ hydrate phase behavior, formation and growth, there is a need to explore other IL families given the vast possibility of IL structural variation and functionalities.

**Figure 1:**
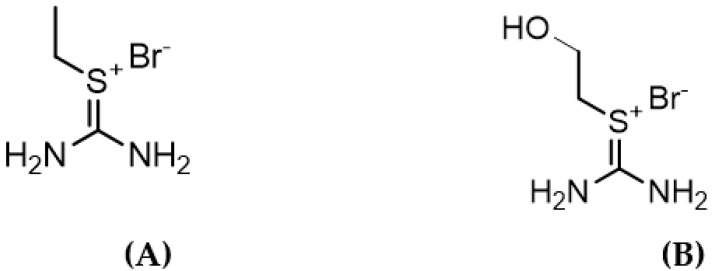
Structures of the ionic liquids used in this work. (**A**) 2-ethylthiouronium bromide and (**B**) 2-(hydroxyethyl)thiouronium bromide.

Recently, it was shown that dimethylsulfoxide could be a replacement for methanol which has been used in the oil/gas industry for decades [42]. It was also emphasized recently that ILs with shorter alkyl chain lengths and containing hydroxyl groups are likely to provide better inhibition results [31]. Therefore, in this work, we have decided to synthesize two thiouronium-based ILs viz., 2-ethylthiouronium bromide [C_2_th][Br] and 2-(hydroxymethyl) thiouronium bromide [C_2_OHth][Br] to test their effect on CO_2_ and THF hydrate formation and dissociation. These ILs have never been used for this purpose. Unlike the cyclic aromatic and non-aromatic ILs tested so far for hydrate inhibition/promotion [31,32,33], thiouronium cation-based ILs have a planar, charge-delocalized acyclic core (Figure 1). These ILs are relatively inexpensive, easy to synthesize and are generally used for metal extraction, peptide coupling, electroplating bath additives, stabilizers for vulcanized rubber, etc. [43,44].

In the present work, we have performed extensive thermodynamic and kinetic testing of the two ILs at different concentrations for CO_2_ and THF hydrate formation and growth using a rocking cell apparatus. It has been reported that this apparatus consumes less energy compared to stirred cell apparatuses while screening the performance of newly developed low-dosage hydrate additives [45]. The reason to choose CO_2_ was because CH_4_ and CO_2_ both form sI type hydrates and CO_2_ poses a relatively low greenhouse effect. An isochoric pressure search method was used to map the phase behavior of CO_2_ hydrates in the presence of ILs. A constant ramping method revealed the hydrate nucleation and dissociation onset temperatures, as well as the growth profiles after nucleation. The growth and morphology of THF hydrates in the presence of ILs at atmospheric pressure provide information about the effectiveness and mode of action of the ILs in altering the growth of sII hydrates—the type of structure formed by natural gas hydrates. The method is very popular to screen the additives due to its rapidness and lower energy consumption [46,47].

## 2. Results and Discussion

### 2.1. Hydrate Phase Behavior

**Figure 2:**
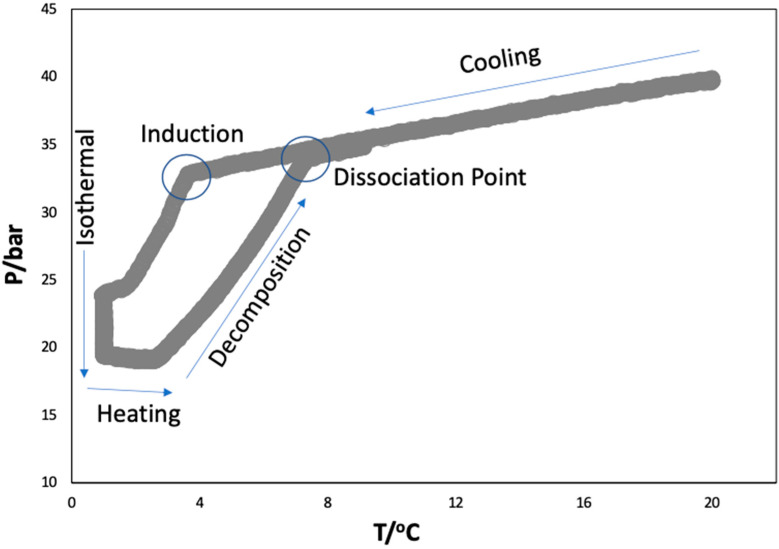
A typical P–T loop obtained for the (CO_2_ + H_2_O) system from the isochoric pressure search method to map the hydrate liquid vapor equilibria.

**Figure 3:**
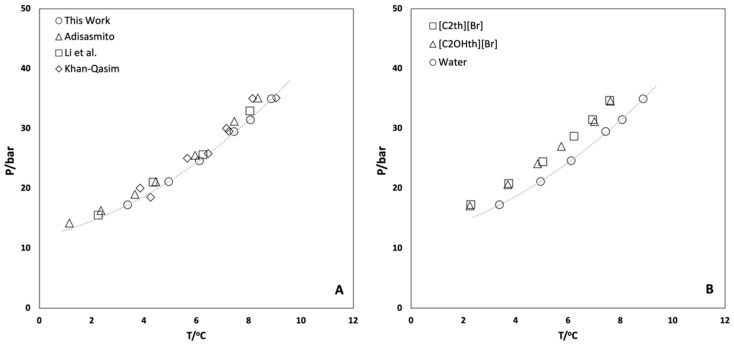
(**A**) CO_2_ hydrate vapor liquid equilibrium (HLVE) conditions in Milli-Q water obtained in this work along with the values reported in the literature for comparison [50,51,52,53] and (**B**) the values obtained in the presence of 10 wt% aqueous solutions of ionic liquids. The dashed lines are just a guide to the eye.

The rocking cell apparatus was validated by obtaining the hydrate dissociation conditions of CO_2_ in Milli-Q water alone before starting the experiments in aqueous solutions of ionic liquids. The hydrate equilibrium experiments were carried out in the pressure range 20–40 bar, following the procedure described in the experimental section and different P–T loops were obtained. From these loops, the dissociation points were calculated by the intersection of the cooling (liquid–vapor) and heating (hydrate–liquid–vapor) equilibrium lines [48,49] (Figure 2). The hydrate dissociation points (P, T) obtained in this work for the well-studied hydrate-forming (CO_2_ + H_2_O) system were used to draw the HLVE curve, which was compared with the data reported in the literature [50,51,52,53] and found to be in good agreement, as shown in Figure 3A.

The hydrate dissociation phenomena in the presence of 10 wt% ionic liquids viz. [C_2_th]Br and [C_2_OHth]Br were studied and the results obtained are plotted in Figure 3B and presented in Table 1. This concentration was chosen to compare the efficiency of the ILs in shifting the equilibrium of hydrate dissociation with classical inhibitors (methanol, glycols) for which data are generally available at 10 wt%. Thermodynamic inhibitors act by preferential hydrogen bonding and altering the water activity that results in the shift of the equilibrium towards lower temperatures and higher pressures. However, it is clear from the results presented in Figure 3B that both ILs are weak inhibitors and show almost similar behavior by shifting the equilibrium towards lower temperatures (~1.2 °C). The addition of a hydroxyl group did not enhance the thermodynamic performance of the studied ILs. However, these results are not surprising since ILs are known for their mild thermodynamic hydrate inhibitory effects and provide shifts typically in the range of 1–3 °C for the equilibrium temperatures [31,32,33] (Figure S1).

### 2.2. Hydrate Dissociation Enthalpies

The dissociation enthalpies of hydrates depend on the host–guest interactions and the water cages around the host that can be altered by the presence of an additive. A significant change in dissociation enthalpies also indicates the participation of the additive in cage formation [54]. The enthalpy of hydrate dissociation in the presence of ILs can be obtained by using the HLVE data points (Table 1) and the Clausius–Clapeyron equation as:

$$\frac{d\left(lnP\right)}{d\left(1/T\right)}=\frac{-\Delta H_{d}}{zR}$$

where *P*, *T*, Δ*H_d_*, z, and *R* are pressure, temperature, dissociation enthalpy, compressibility factor, and gas constant, respectively. The compressibility factor was calculated using the Peng–Robinson equation of state at each set of dissociation conditions presented in Table 1 [32].

The results of the Δ*H_d_* of CO_2_ in H_2_O and in the presence of 10 wt% aqueous IL solutions are presented in Table 1 against each set of dissociation conditions. The values of Δ*H_d_* for CO_2_ are in good agreement with those reported in the literature [55]. It is evident that the Δ*H_d_* values are almost similar in the absence or presence of ILs, thereby indicating that the ILs do not participate in hydrate cage formation.

### 2.3. Hydrate Nucleation Onset Temperatures (T_on_)

**Figure 4:**
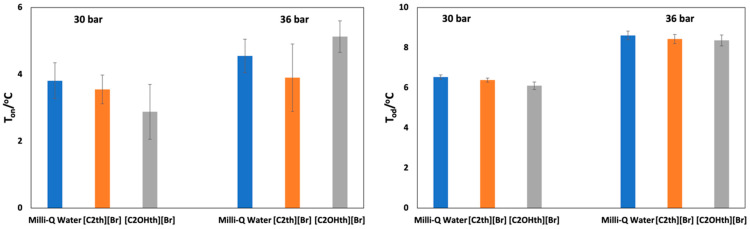
Plots of nucleation (T_on_) (**left**) and decomposition (T_od_) (**right**) onset temperatures for CO_2_ hydrates in Milli-Q water and 1 wt% aqueous ionic liquids solutions at 30 and 36 bar. The error bars represent the standard deviations.

The constant ramping method is a well-perceived method in the literature [56,57] which has been used to obtain the nucleation onset temperatures to screen the efficiency of given kinetic hydrate inhibitors at low concentrations. The results of the nucleation (T_on_) onset temperatures of CO_2_ hydrates in solutions of 1 wt% ILs and Milli-Q water at two different pressures are given in Table 2 along with the standard deviations and plotted in Figure 4 (left). The presented values are an average of 12 measurements for each sample. The confidence intervals (CIs) have also been calculated for the T_on_ at a 95% confidence level using the standard procedure. The CIs at 30 bar can be expressed as T_on_(Milli-Q): 4.1–3.5 °C, T_on_([C_2_th]Br): 3.8–3.3 °C and T_on_([C_2_OHth]Br): 3.3–2.4 °C and at 36 bar it can be expressed as T_on_(Milli-Q): 4.8–4.3 °C, T_on_([C_2_th]Br): 4.5–3.3 °C and T_on_([C_2_OHth]Br): 5.4–4.9 °C.

Repeated runs on the same solutions were carried out to understand if a “memory effect” exist in the absence or presence of ILs [56]. The T_on_ for all the 12 runs is presented in the Supporting Information (Figure S2), which indicate the absence of any memory effect.

A statistical analysis was also conducted on the obtained T_on_ using a *t*-test and the results of t-values and p-values are presented in Table S1. It is clear from the analysis that the values for [C_2_th][Br] at both pressures are not statistically significant, whereas the values are significant for [C_2_OHth][Br].

In the case of 30 bar, the T_on_ in Milli-Q water, [C_2_th][Br] and [C_2_OHth][Br] is 3.8, 3.6 and 2.9 °C, respectively. These results indicate that in the presence of ILs, more subcooling is required for nucleation to occur, more so in the case of [C_2_OHth][Br]. It seems that the presence of an -OH group in the IL increased its kinetic hydrate inhibition performance due to the enhanced possibility of hydrogen bonding with available water. The T_on_ in Milli-Q water, [C_2_th][Br] and [C_2_OHth][Br] is 4.6, 3.9 and 5.1 °C, respectively. These results show the pressure-dependent behavior of these ILs—at low pressure, both the ILs act as inhibitors, whereas at relatively higher pressure (36 bar), [C_2_OHth][Br] promotes hydrate nucleation. However, these results are not surprising as it has been reported elsewhere that the performance of kinetic hydrate inhibitors may deteriorate at high pressures [58], which might be the reason in the present case. Many other substances are reported to work as inhibitors or promoters depending on the working pressure–temperature conditions [59,60]. Therefore, the trends observed for the average T_on_ for fresh and memory solutions at 30 and 36 bar are: [C_2_OHth][Br] < [C_2_th][Br] ≈ Milli-Q water and [C_2_th][Br] ≈ Milli-Q water < [C_2_OHth][Br], respectively. Similar behavior of hydrate nucleation in the presence of additives has been reported elsewhere [57,61].

### 2.4. Hydrate Dissociation Onset Temperatures (T_od_)

The presence of a low-dosage hydrate inhibitor reduces the rate of hydrate formation but does not guarantee that the hydrates will stop forming in the pipelines. The kinetics of hydrate decomposition in the presence of 1 wt% ILs at two aforementioned pressures was also studied using the constant ramping method. The T_od_ at two different pressures is presented in Table 2 along with standard deviations and plotted in Figure 4 (right). The given values are an average of 12 runs. The CIs have also been calculated for the T_od_ at a 95% confidence level using the standard procedure. The CIs at 30 bar can be expressed as T_od_(Milli-Q): 6.6–6.5 °C, T_od_([C_2_th]Br): 6.4–6.3 °C and T_od_([C_2_OHth]Br): 6.1–6.0 °C; and the CIs at 36 bar can be expressed as T_od_(Milli-Q): 8.7–8.6 °C, T_od_([C_2_th]Br): 8.5–8.4 °C and T_od_([C_2_OHth]Br): 8.4–8.3 °C.

Statistical analysis was also conducted on the obtained T_od_ similar to the T_on_ using a *t*-test and the results are presented in Table S1. It is clear that the T_od_ is statistically significant in all cases. As expected, the dissociation point is comparatively more repeatable than the nucleation point [48,62]. At 30 bar, the T_od_ in Milli-Q water, [C_2_th][Br] and [C_2_OHth][Br] is 6.5, 6.4 and 6.1 °C, respectively, whereas, at 36 bar, the values are 8.6, 8.4 and 8.4 °C, respectively (Table 2). Thus, it can be envisaged that, unlike the T_on_, the T_od_ follows similar trends at both pressures, i.e., [C_2_OHth][Br] < [C_2_th][Br] < Milli-Q water. Both the ILs slightly decrease the T_od_ and the trend is in accordance with the results obtained from phase behavior experiments. The higher magnitude of the T_od_ at 36 bar compared to 30 bar is indicative of a large driving force that results in a higher amount of hydrate formation, which requires a relatively higher temperature for decomposition. However, the presence of 1 wt% ILs slightly brought down this temperature.

### 2.5. Hydrate Growth

**Figure 5:**
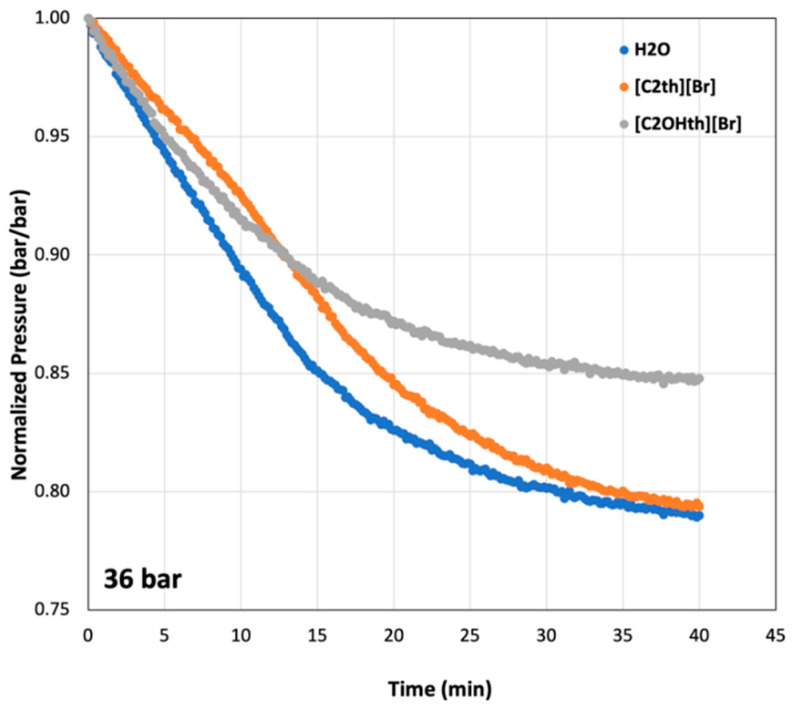
Normalized pressure curves at the initial pressure of 36 bar indicating growth of CO_2_ hydrates with time in 1 wt% aqueous solutions of ionic liquids.

To understand hydrate growth in the presence of the studied ILs, the pressure changes after the T_on_ were normalized (*p*/*p_i_*) and plotted in Figure 5 for the initial pressure of 36 bar. The drop in pressure indicates hydrate formation and their trends inform about relative growth. For instance, after nucleation, in all cases, the hydrates grow to a maximum amount within 40 min (as they were approaching a plateau). The lowest drop was observed in the uninhibited system (Milli-Q water) until reaching a plateau, after which the [C_2_th][Br]-containing system shows more growth. Some additives are known to suppress nucleation but enhance growth compared to a system where the additive is absent [61,63,64]. The system containing [C_2_OHth][Br] showed the lowest levels of hydrate formation throughout the growth step, indicating its potential as an LDHI which suppresses hydrate growth as well.

### 2.6. Hydrate Decomposition

**Figure 6:**
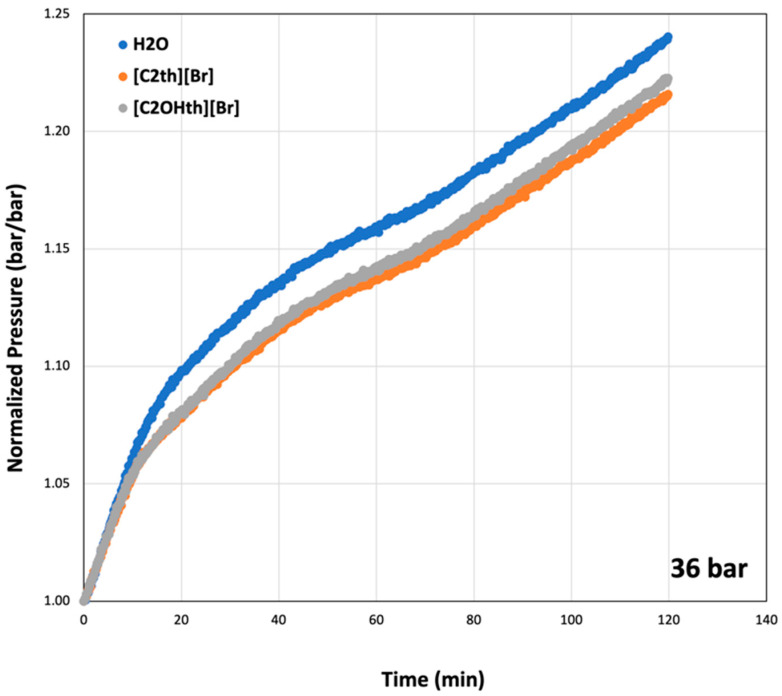
Normalized pressure curves at the initial pressure of 36 bar indicating decomposition of CO_2_ hydrates with time in 1 wt% aqueous solutions of ionic liquids.

To compare hydrate decomposition in the presence of ILs, pressure changes after the T_od_ were normalized (*p*/*p_i_*) and plotted in Figure 6 for the initial pressure of 36 bar. From the plots, one can observe that all the systems follow similar trends and the hydrates were decomposed completely within 2 h. Initially (up to 10 min), all the systems show similar decomposition rates but afterwards, the systems containing ILs shows slightly smaller rates. A closer look at the increase in pressure in each case indicates that the system with Milli-Q water shows the highest increase in pressure. This indicates that more CO_2_ hydrates were produced in the uninhibited systems compared to the systems with ILs. Among ILs, the trends were identical until 40 min. Afterwards, the system containing 1 wt% [C_2_OHth][Br] shows a slightly higher rise in pressure compared to the system containing [C_2_th][Br], indicating more gas released. These are encouraging results as both ILs show not only suppression of nucleation onset and growth, but the hydrates formed in their presence are relatively easy to decompose. Sharifi et al. [65] showed that the synthetic KHIs stabilize the hydrates once they form, which resulted in more gas released and higher decomposition temperatures compared to the uninhibited system. On the contrary, the studied ILs shift decomposition temperatures towards lower values and do not show any sign of hydrate stabilization.

### 2.7. THF Hydrate Growth

**Figure 7:**
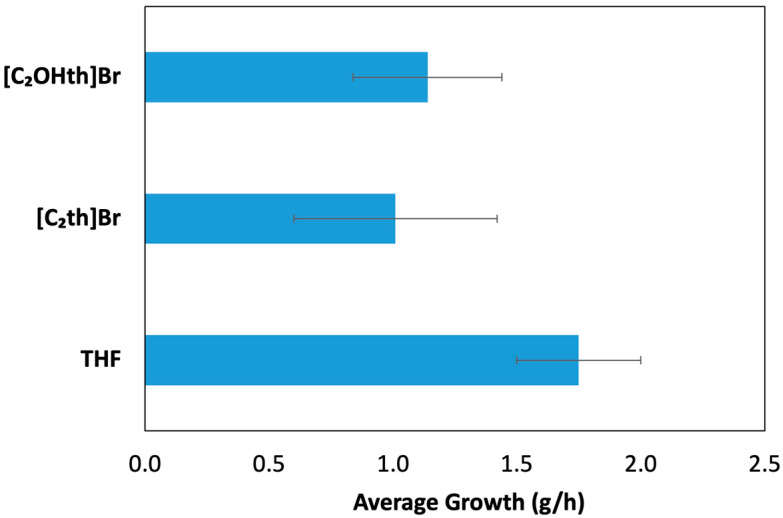
Average growth of THF hydrates (g/h) along with the standard deviations in the absence and presence of thiouronium ILs.

The results of the THF hydrate growth experiment in the absence and presence of ILs are presented in Figure 7 indicating that both ILs suppress the growth of sII hydrates. The trend obtained needs to be further confirmed by testing these ILs for other sII hydrate-forming systems, such as, natural gas mixtures at realistic conditions of high pressure.

**Figure 8:**
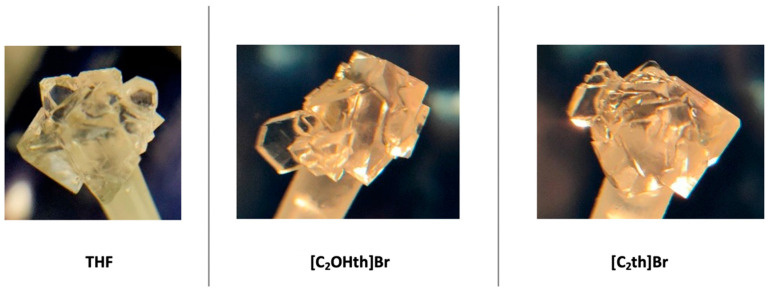
Morphology of THF hydrates in the absence and presence of ILs.

The morphology of the hydrate crystals was also noted in the absence and presence of ILs, and the results are presented in Figure 8. No significant changes in the morphology of THF hydrates were observed [66,67]. In the absence of any additive, the morphology of THF hydrate crystal is regular pyramidal (RP); in the presence of [C_2_OHth]Br, it is RP or occasionally thin plates (TP); in the presence of [C_2_th]Br, it again remains unchanged from RP. This indicates that the ILs are not inhibiting hydrate growth by adsorption at the crystal surface [68]. Rather, it seems that their mode of inhibition is altering the structure of water [69]. Nevertheless, the results are encouraging and show the potential of the ILs as inhibitors for both type sI and sII hydrates.

## 3. Materials and Methods

### 3.1. Materials

Bromoethane (98%), 2-bromoethanol (95%), thiourea (≥99%), acetonitrile (≥99.9%) and ethyl acetate (99%) were purchased from Aldrich (St. Louis, MI, USA) and used as received. CO_2_ with a stated purity of 99.995% was acquired from Alphagaz^TM^ (Air Liquide, Lisboa, Portugal). Milli-Q quality water was used to prepare solutions. A Sartorious balance with a precision of ±1 × 10^−5^ g was used to prepare the test solutions, which were kept in air-tight vials.

### 3.2. Synthesis

The ionic liquids (ILs) used in this work were synthesized and characterized as per the procedure given below.

#### 3.2.1. 2-Ethylthiouronium Bromide (A)

Bromoethane (4.0 equiv.) was added to a stirred solution of thiourea (1.00 equiv.) in 100 mL of acetonitrile in a pressure tube at room temperature. The reaction mixture was then warmed-up to reflux for 3 days. The excess bromoethane and solvent were removed under reduced pressure and the product was dried under vacuum, to give the product without further purification as a white crystalline powder (99% yield) with a purity of ≥99%. ^1^H NMR (400 MHz, DMSO-d6): 1.24 (t, 3H, J = 6.96 Hz, thio-CH_2_CH_3_), 3.18 (q, 2H, J = 6.72 Hz, thio-CH_2_CH_3_), 9.04 (m, 4H, 2 × NH_2_); ^13^C NMR (100 MHz, D_2_O): 14.55, 25.27, 170.36.

#### 3.2.2. 2-(Hydroxyethyl)thiouronium Bromide (B)

2-bromoethanol (1.2 equiv.) was added, in a pressure tube at room temperature, to a stirred solution of thiourea (1.00 equiv.) in 100 mL of acetonitrile. The reaction mixture was then warmed up to reflux for 3 days. The solvent was removed under reduced pressure. The crude product was washed several times with ethyl acetate to remove the 2-bromoethanol excess. The washed product was dried under vacuum to give a white crystalline powder (99% yield) with a purity of ≥99%. ^1^H NMR (400 MHz, DMSO-d6): 3.24 (t, 2H, J = 6.82 Hz, thio-CH_2_CH_2_OH), 3.60–3.68 (m, 2H, thio-CH_2_CH_2_OH), 5.49 (s, 1H, thio-CH_2_CH_2_OH), 9.02 (m, 4H, 2xNH_2_); ^13^C NMR (100 MHz, D_2_O): 34.31, 60.12, 171.13.

### 3.3. Apparatus

A rocking cell apparatus (PSL Systemtechnik, Osterode am Harz, Germany) was used to conduct CO_2_ hydrate formation and dissociation experiments in Milli-Q water and aqueous solutions of the synthesized ionic liquids. Up to 5 stainless steel cells, each with a volume of 40 cm^3^, can be loaded with the desired liquid phase and mounted on top of the rocking axis to perform 5 simultaneous experiments in similar P–T–t conditions. A 17 mm-diameter steel ball was inserted in each cell that rolls back and forth and facilitates mixing. The rocking platform is situated in a bath whose temperature is controlled by a precise thermostat (Huber, Ministat 230, Edison, NJ, USA) while each cell is attached with a separate pressure sensor. The cells can be charged with the gas at a desired pressure using an individual inlet valve for each cell. The software (WinRCS V1.8) provided with the apparatus can be used to define the protocol with desired variables such as temperature ramping, rocking rate, rocking angle and to record the P–T–t data. The cells were filled with 15 mL of Milli-Q water or the aqueous ionic liquid solutions of specific concentration, sealed carefully and mounted on the rocking rig. The air present in the cells was replaced by flushing the test gas (CO_2_) 2–3 times. Afterwards, the cells were charged with the CO_2_ at a given pressure as required for the experiment. The detailed description of the rocking cell apparatus can be found elsewhere [45,56,70]. Different methods were used to study the phase behavior, induction time and formation and dissociation kinetics of hydrates in Milli-Q water and in aqueous solutions of ionic liquids as described below.

### 3.4. Hydrate Phase Equilibria

An isochoric pressure search method was used to obtain the hydrate phase equilibrium data. After loading the samples, the temperature of the bath was set to 20 °C and the CO_2_ was injected to obtain the desired initial pressure in each cell. The system was left with rocking for a period of 1 h to ensure gas dissolution and stabilization at the desired temperature and pressure. Afterwards, the system was cooled from 20 to 1 °C along 6 h at a cooling rate of 3.17 °C/h, which resulted in the nucleation of the gas hydrates. The system was left isothermal at 1 °C for a period of 12 h for the hydrates to grow. Later, the system was heated from 1 to 20 °C with a very slow rate of 0.3 °C/h to dissociate the formed hydrates. This heating rate is adequate to obtain good quality hydrate dissociation points [62,71]. The cells were rocked at an angle of 45° and a rate of 10 rocks/min throughout the experiments. Typical P–T loops as described in Figure 2 were obtained from which the hydrate dissociation points were calculated as described elsewhere [48] and presented in Table 1.

### 3.5. Hydrate Nucleation and Dissociation Onset Temperatures

**Figure 9:**
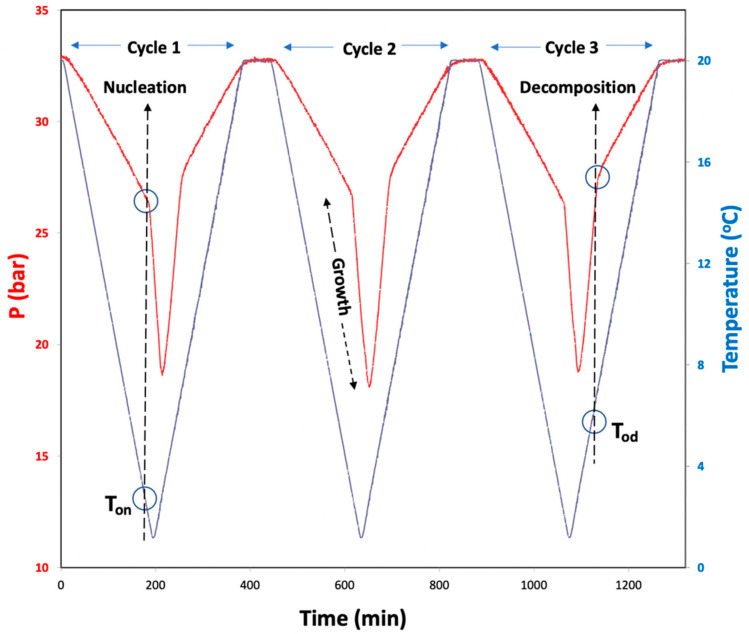
A scheme of constant ramping method cycles and typical P–T and T–t curves obtained for the (CO_2_ + H_2_O) system. The hydrate nucleation onset temperature (T_on_), growth and dissociation onset temperature (T_od_) are indicated on the curves.

A constant ramping method with continuous cooling and heating segments throughout the experiment was employed to determine hydrate formation (T_on_) and dissociation onset temperatures (T_od_) for the systems with Milli-Q water and 1 wt% aqueous solutions of ionic liquids, as depicted in Figure 9. A comparison of these values provides information about the performance of the ILs. This method mimics the field conditions more closely compared to isothermal-isochoric ones [49]. The cells were loaded with 15 mL test solution and mounted on the rocking axis as described before. The cells were then purged a couple of times with 5–10 bars of CO_2_ to replace the air present in the cell. Later, the CO_2_ was introduced into each cell individually at a desired initial pressure. The system was equilibrated at 20 °C for an hour while rocking. Further, the bath was cooled from 20 to 1 °C with a rate of 0.1 °C/min and heated from 1 to 20 °C with a similar rate. Throughout the experiments, the cells were rocked at an angle of 45° with a rate of 10 rocks/min. The P–T–t data were collected using the commercial software. The pressure–temperature changes over time for a typical constant ramping experiment are depicted in Figure 9. From these P–t and corresponding T–t curves, nucleation (T_on_) and dissociation (T_od_) onset temperatures can be identified with an abrupt change in pressure, as represented in Figure 9. After completion of a cooling–heating cycle, the system was allowed to reach initial P–T conditions; and after 60 min, another cooling–heating cycle began and the obtained values for the second cycle are presented. A third cycle, after 60 min equilibration was also performed in a similar manner. Each sample was tested in similar conditions for at least 4 times and average values of the 12 experiments are reported in Table 2. More information about this method can be found elsewhere [49,56].

### 3.6. THF Hydrate Growth

**Figure 10:**
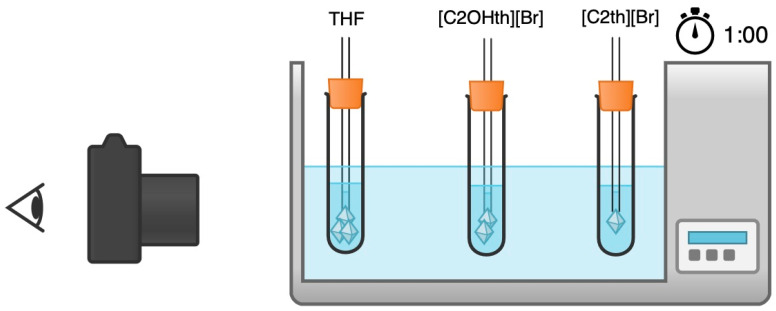
Schematic representation of the screening experiments for the ILs as THF hydrate crystal growth inhibitors. The figure was drawn using the free version of online Chemix^®^ software.

It is well known that a 19.1 wt% aqueous solution of THF forms structure II types of hydrates at atmospheric pressure, which are structurally similar to natural gas hydrates [46,47]. Therefore, this method is used frequently for initial screening of the additives used for controlled gas hydrate formation. THF hydrate growth at atmospheric pressure and at a temperature of −0.5 °C was assessed in the absence and presence of the ILs. A test solution of 1:17 ratio of THF:H_2_O was prepared with 3.6 wt% NaCl added to it. It was used as the stock/reference solution and was used to prepare 1 wt% IL solutions for screening. Specially designed glass cells were filled with test solutions and were cooled to −0.5 °C in a highly accurate (±0.05 °C) cooling bath. As soon as the solutions reached thermal equilibrium, hollow glass tubes with a small ice crystal attached to it were inserted in each cell to initiate nucleation. The growth of the THF hydrate crystal per hour was measured by weighing the crystals formed as depicted in Figure 10. At least 4–6 experiments were performed, and an average value along with the standard deviations has been reported. The morphology of the crystals in the absence and presence of ILs was also recorded using a camera and reported. More details about the method can be found elsewhere [66,67,72].

## 4. Conclusions

Two thiouronium ILs were synthesized and tested for their thermodynamics and kinetic inhibition/promotion behavior towards CO_2_ and THF hydrates. Hydrate equilibrium measurements indicated that both the ILs act as thermodynamic hydrate inhibitors at 10 wt% by suppressing the hydrate equilibrium ~1.2 °C. The constant ramping method that mimics the field conditions carried out at 1 wt% concentration of ILs revealed that the effect is pressure dependent. At 30 bar, both ILs act as inhibitors by decreasing the hydrate nucleation onset temperature compared to the uninhibited system. Statistical analysis revealed that the effect of [C_2_th][Br] on nucleation was not significant, whereas, at 36 bar, [C_2_OHth] [Br] exhibits a hydrate promotion effect. [C_2_OHth] [Br] suppressed the hydrate nucleation onset temperature at the studied pressures, indicating that more subcooling is required to form the hydrates. Normalized pressure curves after the nucleation and inhibition onset temperatures indicate that the ILs also suppress growth after nucleation, which resulted in less gas released during decomposition. Therefore, thermodynamic and kinetic studies show that both thiouronium ILs act as dual-function hydrate inhibitors that not only shift the equilibrium towards lower temperatures but also suppress hydrate formation and growth. The THF hydrate growth experiments performed at atmospheric pressure indicate that the ILs can also inhibit the growth of natural gas hydrates (sII) and their mode of action is not adsorption at the crystal surface but altering the structure of water. To conclude, experimental methodology, concentration and pressure-temperature range are significant when screening for a low-dosage hydrate additive.

## Figures and Tables

**Table 1:** CO_2_ hydrate dissociation conditions and enthalpies (Δ*H_d_*) in water and in 10 wt% aqueous solutions of ionic liquids.

Milli-Q Water			[C_2_th][Br]			[C_2_OHth][Br]		
T/°C	P/bar	Δ*H_d_*/kJ·mol^−1^	T/°C	P/bar	Δ*H_d_*/kJ·mol^−1^	T/°C	P/bar	Δ*H_d_*/kJ·mol^−1^
3.38	17.23	73.5	2.28	17.26	73.4	2.25	17.05	73.0
4.95	21.10	71.1	3.73	20.78	71.2	3.70	20.60	70.9
6.12	24.58	68.9	5.04	24.42	68.9	4.84	24.08	68.7
7.44	29.46	65.7	6.23	28.67	66.1	5.74	27.01	66.8
8.07	31.44	64.4	6.94	31.43	64.2	7.01	31.15	64.0
8.87	34.94	61.9	7.59	34.66	61.6	7.62	34.54	61.5

**Table 2:** Average nucleation (T_on_) and dissociation (T_od_) onset temperatures for Milli-Q water and 1 wt% aqueous ionic liquids solutions obtained from 12 experiments for each data point along with standard deviations using the constant ramping method.

	P = 30 bar		P = 36 bar	
System	T_on_/°C	T_od_/°C	T_on_/°C	T_od_/°C
Milli-Q Water	3.8 ± 0.5	6.5 ± 0.1	4.6 ± 0.5	8.6 ± 0.2
[C_2_th][Br]	3.6 ± 0.4	6.4 ± 0.1	3.9 ± 1.0	8.4 ± 0.2
[C_2_OHth][Br]	2.9 ± 0.8	6.1 ± 0.2	5.1 ± 0.5	8.4 ± 0.3

## Data Availability

Not applicable.

## Supplementary Materials

The following supporting information can be downloaded at: https://www.mdpi.com/article/10.3390/ijms23063292/s1.

## References

[1] Koh C.A., Sloan E.D., Sum A.K., Wu D.T. (2011). Fundamentals and Applications of gas hydrates. Annu. Rev. Chem. Biomol. Eng..

[2] Hassanpouryouzband A., Joonaki E., Farahani M.V., Takeya S., Ruppel C., Yang J., English N.J., Schicks J.M., Edlmann K., Mehrabian H. (2020). Gas hydrates in sustainable chemistry. Chem. Soc. Rev..

[3] Ripmeester J.A., Alavi S. (2016). Some current challenges in clathrate hydrate science: Nucleation, decomposition and the memory effect. Curr. Opin. Solid State Mater. Sci..

[4] Sum A.K., Koh C.A., Sloan E.D. (2009). Clathrate Hydrates: From Laboratory Science to Engineering Practice. Ind. Eng. Chem. Res..

[5] Sabil K.M., Partoon B. (2018). Recent advances on carbon dioxide capture through a hydrate-based gas separation process. Curr. Opin. Green Sust. Chem..

[6] Veluswamy H.P., Kumar A., Seo Y., Lee J.D., Linga P. (2018). A review of solidified natural gas (SNG) technology for gas storage via clathrate hydrates. Appl. Energy.

[7] Eslamimanesh A., Mohammadi A.H., Richon D., Naidoo P., Ramjugernath D. (2012). Application of gas hydrate formation in separation processes: A review of experimental studies. J. Chem. Thermodyn..

[8] Straume E.O., Morales R.E.M., Sum A.K. (2019). Perspectives on Gas Hydrates Cold Flow Technology. Energy Fuels.

[9] Babu P., Nambiar A., He T., Karimi I.A., Lee J.D., Englezos P., Linga P. (2018). A Review of Clathrate Hydrate Based Desalination to Strengthen Energy–Water Nexus. ACS Sustain. Chem. Eng..

[10] Babu P., Linga P., Kumar R., Englezos P. (2015). A review of the hydrate-based gas separation (HBGS) process for carbon dioxide pre-combustion capture. Energy.

[11] Sloan E.D., Koh C.A. (2008). Clathrate Hydrates of Natural Gases.

[12] Giavarini C., Hester K. (2011). Gas Hydrate: Immense Energy Potential and Environmental Challenges.

[13] Sum A.K., Koh C.A., Sloan E.D. (2012). Developing a Comprehensive Understanding and Model of Hydrate in Multiphase Flow: From Laboratory Measurements to Field Applications. Energy Fuels.

[14] Fan S.-S., Chen G.-J., Ma Q.-L., Guo T.-M. (2000). Experimental and modeling studies on the hydrate formation of CO_2_ and CO_2_-rich gas mixtures. Chem. Eng. J..

[15] Nasir Q., Lau K.K., Lal B., Sabil K.M. (2014). Hydrate dissociation condition measurement of CO_2_—Rich mixed gas in the presence of methanol/ethylene glycol and mixed methanol/ethylene glycol + electrolyte aqueous solution. J. Chem. Eng. Data.

[16] Creek J.L. (2012). Efficient Hydrate Plug Prevention. Energy Fuels.

[17] Lafond P.G., Olcott K.A., Sloan E.D., Koh C.A., Sum A.K. (2012). Measurements of methane hydrate equilibrium in systems inhibited with NaCl and methanol. J. Chem. Thermodyn..

[18] Kelland M.A. (2006). History of the development of low dosage hydrate inhibitors. Energy Fuels.

[19] Roosta H., Dashti A., Mazloumi S.H., Varaminian F. (2016). Inhibition properties of new amino acids for prevention of hydrate formation in carbon dioxide-water system: Experimental and modeling investigations. J. Mol. Liq..

[20] Esperança J.M., Tariq M., Pereiro A.B., Araújo J.M., Seddon K.R., Rebelo L.P.N. (2019). Anomalous and Not-So-Common Behaviour in Common Ionic Liquids and Ionic Liquid-containing Systems. Front. Chem..

[21] Tariq M., Shimizu K., Esperança J.M.S.S., Canongia Lopes J.N., Rebelo L.P.N. (2015). Viscosity minima in binary mixtures of ionic liquids + molecular solvents. Phys. Chem. Chem. Phys..

[22] Petkovic M., Seddon K.R., Rebelo L.P.N., Pereira C.S. (2011). Ionic liquids: A pathway to environmental acceptability. Chem. Soc. Rev..

[23] Petkovic M., Ferguson J.L., Gunaratne H.Q.N., Ferreira R., Leitao M.C., Seddon K.R., Rebelo L.P.N., Pereira C.S. (2010). Novel biocompatible cholinium-based ionic liquids—Toxicity and biodegradability. Green Chem..

[24] Weingärtner H., Cabrele C., Herrmann C. (2012). How ionic liquids can help to stabilize native proteins. Phys. Chem. Chem. Phys..

[25] Takekiyo T., Yoshimura Y. (2018). Suppression and dissolution of amyloid aggregates using ionic liquids. Biophys. Rev.

[26] Bera A., Belhaj H. (2016). Ionic liquids as alternatives of surfactants in enhanced oil recovery—A state-of-the-art review. J. Mol. Liq..

[27] Zhang Q.B., Hua Y.X. (2009). Corrosion inhibition of mild steel by alkylimidazolium ionic liquids in hydrochloric acid. Electrochim. Acta.

[28] Ibrahim M.H., Hayyan M., Hashim M.A., Hayyan A. (2017). The role of ionic liquids in desulfurization of fuels: A review. Renew. Sustain. Energ. Rev..

[29] Ferreira T.J., Vera A.T., de Moura B.A., Esteves L.M., Tariq M., Esperança J., Esteves I. (2020). Paramagnetic Ionic Liquid/Metal Organic Framework Composites for CO_2_/CH_4_ and CO_2_/N_2_ Separations. Front. Chem..

[30] Lee W., Shin J.-Y., Kim K.-S., Kang S.-P. (2016). Kinetic Promotion and Inhibition of Methane Hydrate Formation by Morpholinium Ionic Liquids with Chloride and Tetrafluoroborate Anions. Energy Fuels.

[31] Tariq M., Rooney D., Othman E., Aparicio S., Atilhan M., Khraisheh M. (2014). Gas hydrate inhibition: A review of the role of ionic liquids. Ind. Eng. Chem. Res..

[32] Bavoh C.V., Nashed O., Rehman A.N., Othman N.A.A.B., Lal B., Sabil K.M. (2021). Ionic liquids as gas hydrate thermodynamic inhibitors. Ind. Eng. Chem. Res..

[33] Haq I.U., Qasim A., Lal B., Zaini D.B., Foo K.S., Mubashir M., Khoo K.S., Vo D.-V.N., Leroy E., Show P.L. (2022). Ionic liquids for the inhibition of gas hydrates. A review. Env. Chem. Lett..

[34] Makino T., Matsumoto Y., Sugahara T., Ohgaki K., Masuda H. Effect of Ionic Liquid on Hydrate Formation Rate in Carbon Dioxide Hydrates. Proceedings of the 7th International Conference on Gas Hydrates (ICGH).

[35] Cha J.H., Ha C., Kang S.P., Kang J.W., Kim K.S. (2016). Thermodynamic Inhibition of CO_2_ Hydrate in the Presence of Morpholinium and Piperidinium Ionic Liquids. Fluid Phase Equilib..

[36] Khan M.S., Lal B., Bavoh C.B., Keong L.K., Bustam A., Bt Mellon N. (2017). Influence of ammonium based compounds for gas hydrate mitigation: A short review. Indian J. Sci. Tech..

[37] Khan M.S., Partoon B., Bavoh C.B., Lal B., Mellon N.B. (2017). Fluid Phase Equilibria Influence of Tetramethylammonium Hydroxide on Methane and Carbon Dioxide Gas Hydrate Phase Equilibrium Conditions. Fluid Phase Equilib..

[38] Khan M.S., Bavoh C.B., Partoon B., Nashed O., Lal B., Bt Mellon N. (2018). Impacts of ammonium based ionic liquids alkyl chain on thermodynamic hydrate inhibition for carbon dioxide rich binary gas. J. Mol. Liq..

[39] Khan M.S., Bavoh C.B., Lal B., Bustam A.M. (2018). Kinetic assessment of tetramethyl ammonium hydroxide (ionic liquid) for carbon dioxide methane binary mix gas hydrates. Recent Advances in Ionic Liquids.

[40] Bavoh C.B., Lal B., Keong L.K. (2016). Synergic kinetic inhibition effect of EMIM-Cl+PVP on CO_2_ hydrate formation. Procedia Eng..

[41] Chun L.K., Jafar A. (2013). Ionic liquid as a low dosage hydrate inhibitor for flow assurance in pipeline. Asian J. Sci. Res..

[42] Semenov A.P., Mendegaziev R.I., Stoporev A.S., Istomin V.A., Sergeeva D.V., Ogienko A.G., Vinokurov V.A. (2021). The pursuit of a more powerful thermodynamic hydrate inhibitor than methanol. Dimethylsulfoxide as a case study. Chem. Eng. J..

[43] Abai M., Holbrey J.D., Rogers R.D., Srinivasan G. (2010). Ionic liquid S-alkylthiouronium salts. New J. Chem..

[44] Torriero A.A.J., Siriwardana A.I., Bond A.M., Burgar I.M., Dunlop N.F., Deacon G.B., MacFarlane D.R. (2009). Physical and Electrochemical Properties of Thioether-Functionalized Ionic Liquids. J. Phys. Chem. B.

[45] Foroutan S., Mohsenzade H., Dashti A., Roosta H. (2021). New insights into the evaluation of kinetic hydrate inhibitors and energy consumption in rocking and stirred cells. Energy.

[46] Makogon T.Y., Larsen R., Knight C.A., Sloan E.D. (1997). Melt growth of tetrahydrofuran clathrate hydrate and its inhibition: Method and first results. J. Cryst. Growth.

[47] Larsen R., Knight C.A., Sloan E.D. (1998). Clathrate hydrate growth and inhibition. Fluid Phase Equilibria.

[48] Tohidi B., Burgass R.W., Danesh A., Østergaard K.K., Todd A.C. (2000). Improving the Accuracy of Gas Hydrate Dissociation Point Measurements. Annal. N. Y. Acad. Sci..

[49] Mu L., Solms N.V. (2020). Inhibition of natural gas hydrate in the system containing salts and crude oil. J. Pet. Sci. Eng..

[50] Adisasmito S., Frank III R.J., Sloan E.D. (1991). Hydrates of carbon dioxide and methane mixtures. J. Chem. Eng. Data.

[51] Li S., Fan S., Wang J., Lang X., Wang Y. (2010). Semiclathrate Hydrate Phase Equilibria for CO_2_ in the Presence of Tetra-n-butyl Ammonium Halide (Bromide, Chloride, or Fluoride). J. Chem. Eng. Data.

[52] Khan M.S., Bavoh C.B., Partoon B., Lal B., Bustam M.A., Shariff A.M. (2017). Thermodynamic effect of ammonium based ionic liquids on CO_2_ hydrates phase boundary. J. Mol. Liq..

[53] Qasim A., Khan M.S., Lal B., Shariff A.M. (2019). Phase equilibrium measurement and modeling approach to quaternary ammonium salts with and without monoethylene glycol for carbon dioxide hydrates. J. Mol. Liq..

[54] Tariq M., Connor E., Thompson J., Khraisheh M., Atilhan M., Rooney D. (2016). Doubly dual nature of ammonium-based ionic liquids for methane hydrates probed by rocking-rig assembly. RSC Adv..

[55] Sabil K.M., Witkamp G.-J., Peters C.J. (2010). Estimations of enthalpies of dissociation of simple and mixed carbon dioxide hydrates from phase equilibrium data. Fluid Phase Equilib..

[56] Pandey J.S., Daas Y.J., Solms N.v. (2020). Screening of Amino Acids and Surfactant as Hydrate Promoter for CO_2_ Capture from Flue Gas. Processes.

[57] Mu L., Ramløv H., Søgaard T.M.M., Jørgensen T., de Jongh W.A., von Solms N. (2019). Inhibition of methane hydrate nucleation and growth by an antifreeze protein. J. Petro. Sci. Eng..

[58] Makogon T.Y., Makogon T.Y. (2019). Chapter 5—Flow restrictions and blockages in operations. Handbook of Multiphase Flow Assurance.

[59] Al-Adel S., Dick J.A.G., El-Ghafari R., Servio P. (2008). The effect of biological and polymeric inhibitors on methane gas hydrate growth kinetics. Fluid Phase Equilib..

[60] Chen Q., Yu Y., Zeng P., Yang W., Liang Q., Peng X., Liu Y., Hu Y. (2008). Effect of 1-butyl-3-methylimidazolium tetrafluoroborate on the formation rate of CO_2_ hydrate. J. Nat. Gas Chem..

[61] Daraboina N., Malmos C., von Solms N. (2013). Synergistic kinetic inhibition of natural gas hydrate formation. Fuel.

[62] Tariq M., Soromenho M.R.C., Rebelo L.P.N., Esperanca J.M.S.S. (2021). Insights into CO_2_ hydrates formation and dissociation at isochoric conditions using a rocking cell apparatus. Chem. Eng. Sci..

[63] Lederhos J.P., Long J.P., Sum A., Christiansen R.L., Sloan E.D. (1996). Effective kinetic inhibitors for natural gas hydrates. Chem. Eng. Sci..

[64] Perfeldt C.M., Chua P.C., Daraboina N., Friis D., Kristiansen E., Ramløv H., Woodley J.M., Kelland M.A., Solms N.V. (2014). Inhibition of gas hydrate nucleation and growth: Efficacy of an antifreeze protein from the longhorn beetle *Rhagium mordax*. Energy Fuels.

[65] Sharifi H., Ripmeester J., Walker V.K., Englezos P. (2014). Kinetic inhibition of natural gas hydrates in saline solutions and heptane. Fuel.

[66] Sefidroodi H., Chua P.C., Kelland M.A. (2011). THF hydrate crystal growth inhibition with small anionic organic compounds and their synergistic properties with the kinetic hydrate inhibitor poly(N-vinylcaprolactam). Chem. Eng. Sci..

[67] Bak I.G., Heo C.-H., Kelland M.A., Lee E., Kang B.-G., Lee J.-S. (2021). Clathrate hydrate inhibition by polyisocyanate with diethylammonium group. Langmuir.

[68] Villano D.L., Kelland M.A. (2010). An investigation into the kinetic hydrate inhibitor properties of two imidazolium-based ionic liquids on structure II gas hydrate. Chem. Eng. Sci..

[69] Sa J.-H., Kwak G.-H., Han K., Ahn D., Cho S.J., Lee J.D., Lee K.-H. (2016). Inhibition of methane and natural gas hydrate formation by altering the structure of water with amino acids. Sci. Rep..

[70] Altamash T., Qureshi M.F., Aparicio S., Aminnaji M., Tohidi B., Atilhan M. (2017). Gas hydrates inhibition via combined biomolecules and synergistic materials at wide process conditions. J. Nat. Gas Sci. Eng..

[71] Semenov A.P., Medvedev V.I., Gushchin P.A., Yakushev V.S. (2015). Effect of heating rate on the accuracy of measuring equilibrium conditions for methane and argon hydrates. Chem. Eng. Sci..

[72] Reza J., Trejo A., Rebolledo-Liberos M.E., Guzman-Lucero D. (2018). Inhibition of structure II hydrates formation by salt-tolerant N-vinyl lactam-based terpolymers. J. Nat. Gas Sci. Eng..

