# Supplementary material for: Effect of Thiouronium-Based Ionic Liquids on the Formation and Growth of CO2 (sI) and THF (sII) Hydrates
Source: Int J Mol Sci. 2022 Mar 18;23(6):3292. doi: 10.3390/ijms23063292 (PMC8955390; doi:10.3390/ijms23063292)
Supplement: Supplementary file 1 [file ijms-23-03292-s001.zip › ijms-1591947-supplementary.pdf]

Supporting Information

Effect of Thiouronium-based Ionic Liquids on the Formation and Growth of CO_2 (sI) and THF (sII) Hydrates

Mário R. C. Soromenho, Anastasiia Keba, José M. S. S. Esperança,* Mohammad Tariq*

LAQV, REQUIMTE, Departamento de Química,
Faculdade de Ciências e Tecnologia,
Universidade NOVA de Lisboa, 2829-516 Caparica, Portugal
E-mails: jmesp@fct.unl.pt (JMSSE); tariq@fct.unl.pt (MT),

Figure S1. Comparison of CO_2 hydrates phase equilibria in presence of ~10 wt% ILs reported in the literature [1-5] along with the two thiouronium ionic liquids tested in this work. The dotted line represents the CO_2 hydrates phase equilibria in absence of any additive.

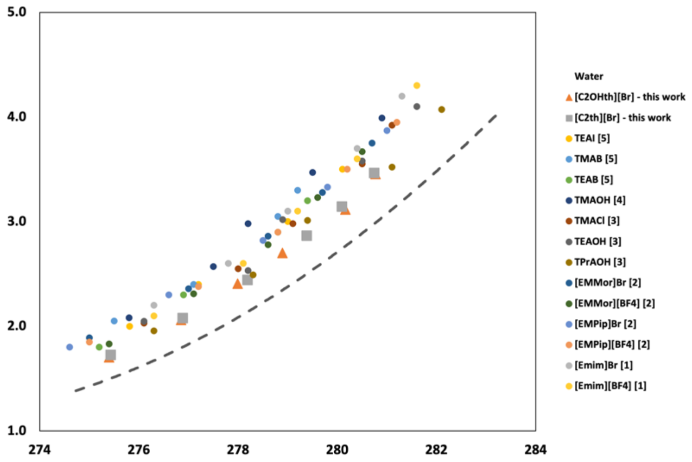

Figure S2. Onset nucleation temperatures (T_{on}) of CO_2 hydrates in Milli-Q water and in 1 wt% aqueous solution of thiouronium ionic liquids at 30 bar and 36 bar obtained from 12 different experiments (represented at x-axis). The blue dashed lines represent the average T_{on} values for the system and are reported in Table 2. The error bars represent the standard deviation values of the measurements for respective set of experiment.

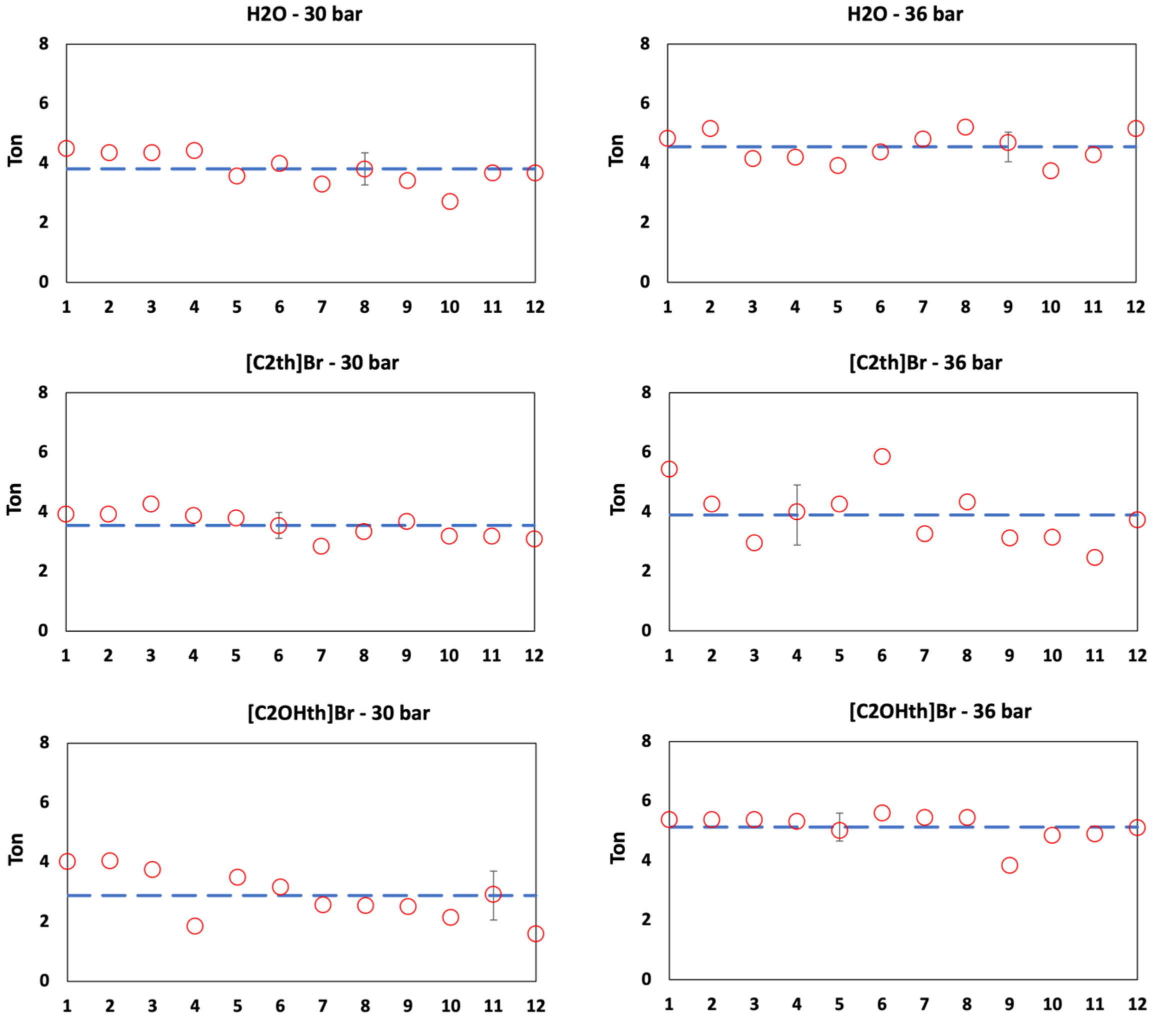

Table S1. Results of the twin-tailed t-test of the onset temperature of nucleation (T_{on}) and decomposition temperatures (T_{od}) values for the studied (H_2O+CO_2) and (H_2O+CO_2+IL) systems obtained at different pressures.

Pressure (P/bar)	Milli Q water – [C_2th][Br]	Milli Q water – [C_2OHth][Br]
**T_{on} Values**		
30	t-value: 1.29871<br>P-value: 0.207493<br>Remarks: result not significant at P<.05	t-value: 3.26739<br>P-value: 0.003524<br>Remarks: result significant at P<.05
36	t-value: 1.98456<br>P-value: 0.059804<br>Remarks: result not significant at P<.05	t-value: -2.94431<br>P-value: 0.007501<br>Remarks: result significant at P<.05
**T_{od} Values**		
30	t-value: 5.01324<br>P-value: 0.000051<br>Remarks: result significant at P<.05	t-value: 8.20428<br>P-value: <0.00001<br>Remarks: result significant at P<.05
36	t-value: 2.2334<br>P-value: 0.036013<br>Remarks: result significant at P<.05	t-value: 2.81639<br>P-value: 0.010054<br>Remarks: result significant at P<.05

References:

[1] Makino, T.; Matsumoto, Y.; Sugahara, T.; Ohgaki, K.; Masuda, H. Effect of Ionic Liquid on Hydrate Formation Rate in Carbon Dioxide Hydrates. In Proceedings of the 7th International Conference on Gas Hydrates (ICGH); Edinburgh, Scotland, United Kingdom, 2011; pp 2−5.

[2] Cha, J. H.; Ha, C.; Kang, S. P.; Kang, J. W.; Kim, K. S. Thermodynamic Inhibition of CO2 Hydrate in the Presence of Morpholinium and Piperidinium Ionic Liquids. Fluid Phase Equilib. 2016, 413, 75−79.

[3] Khan, M. S.; Bavoh, C. B.; Partoon, B.; Lal, B.; Bustam, M. A.; Shariff, A. M. Thermodynamic Effect of Ammonium Based Ionic Liquids on CO_2 Hydrates Phase Boundary. J. Mol. Liq. 2017, 238, 533.

[4] Khan, M. S.; Partoon, B.; Bavoh, C. B.; Lal, B.; Mellon, N. B. Fluid Phase Equilibria Influence of Tetramethylammonium Hydroxide on Methane and Carbon Dioxide Gas Hydrate Phase Equilibrium Conditions. Fluid Phase Equilib. 2017, 440, 1−8.

[5] Qasim, A.; Khan, M. S.; Lal, B.; Shariff, A. M. Phase Equilibrium Measurement and Modeling Approach to Quaternary Ammonium Salts with and without Monoethylene Glycol for Carbon Dioxide Hydrates. J. Mol. Liq. 2019, 282, 106−114.
